# A modified approach for closing endoscopic submucosal dissection defects using clip with line pulley securing technique and endoloop

**DOI:** 10.1016/j.vgie.2024.03.014

**Published:** 2024-03-27

**Authors:** Yohei Minato, Ken Ohata, Yoshiaki Kimoto, Shunya Takayanagi, Yuki Kano, Deepak Madhu, Hideyuki Chiba, Makoto Kobayashi

**Affiliations:** 1 Department of Gastrointestinal Endoscopy, NTT Medical Center Tokyo, Tokyo, Japan; 2 Department of Gastroenterology, Omori Red Cross Hospital, Tokyo, Japan; 3 Department of Gastroenterology, Yokkaichi Municipal Hospital, Mie, Japan

## Introduction

Endoscopic submucosal dissection (ESD) has been developed as a minimally invasive treatment. However, in some instances, closure of post-ESD defects may be necessary in patients with high risk of delayed bleeding, or in patients with deep muscle injury or intra-procedural perforation. Although hemostatic clips may be used to close small defects, closure of larger defects is challenging. Purse-string closure method is suitable for larger defects but may require double-channel scope, scope replacement, and poses alignment complexities.^1^ Suturing devices are costly and cumbersome.^2^^,^^3^ Technique selection should consider defect size, procedural ease, equipment accessibility, and cost-effectiveness.

We had reported the clip with line pulley suturing (CLiPS) technique using handcrafted modified anchoring clip (CLiPS 1.0)^4^ or plastic detachable snare (CLiPS 2.0).^5^ Although CLiPS 1.0 required manual modification of clips to act as a knot, CLiPS 2.0 required use of 2 lines. Here, we have further modified the CLiPS technique using a plastic detachable snare with only 1 line (CLiPS 3.0).

**Figure 1:**
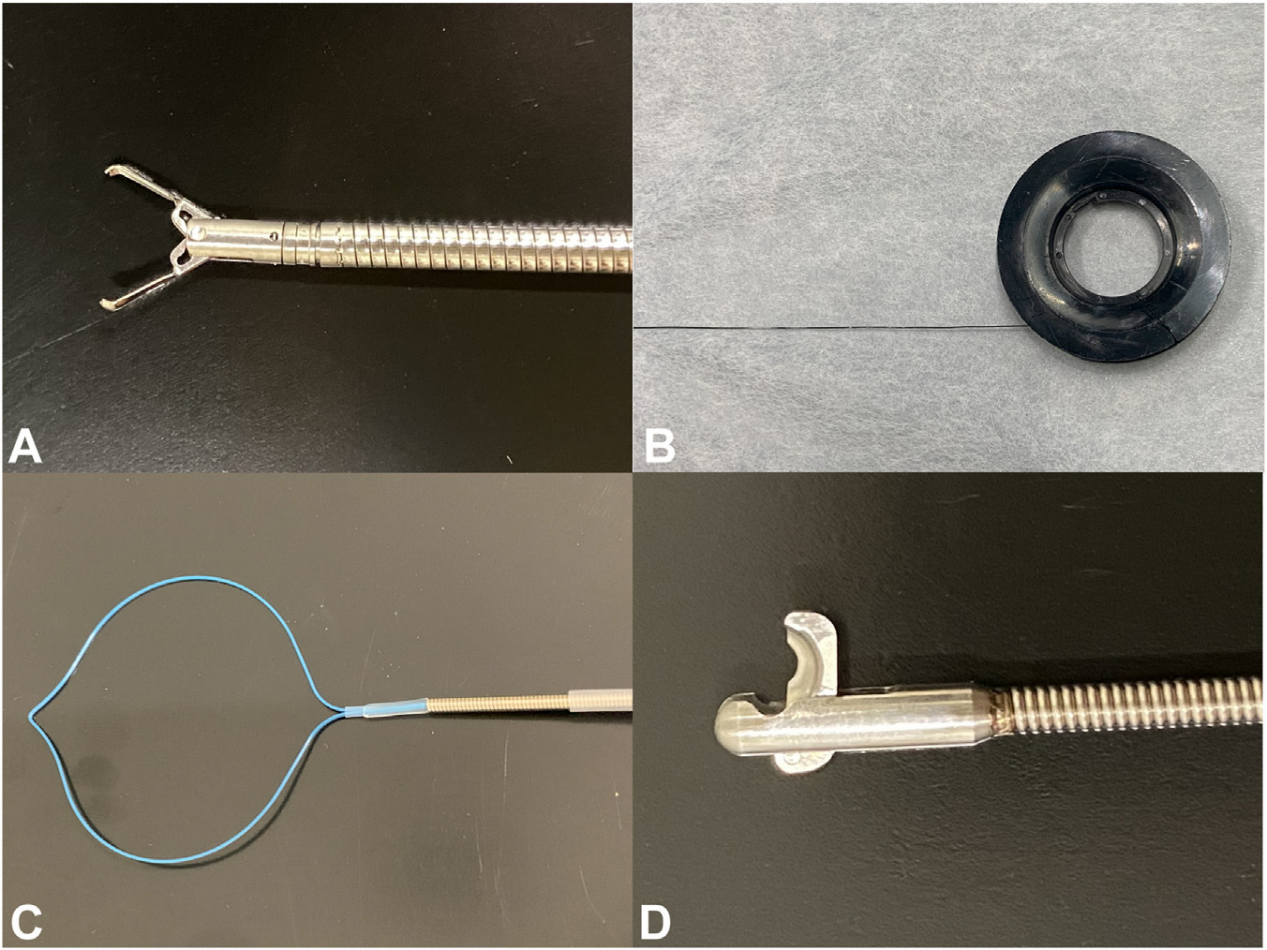
Devices required for clip with line pulley suturing technique. **A,** Sure Clip (Micro-Tech, Nanjing, China). **B,** Bear nylon monofilament suture (BEAR Medic, Ibaraki, Japan). **C,** Polyloop (Olympus, Tokyo, Japan). **D,** FS-5L-1 (Olympus).

**Figure 2:**
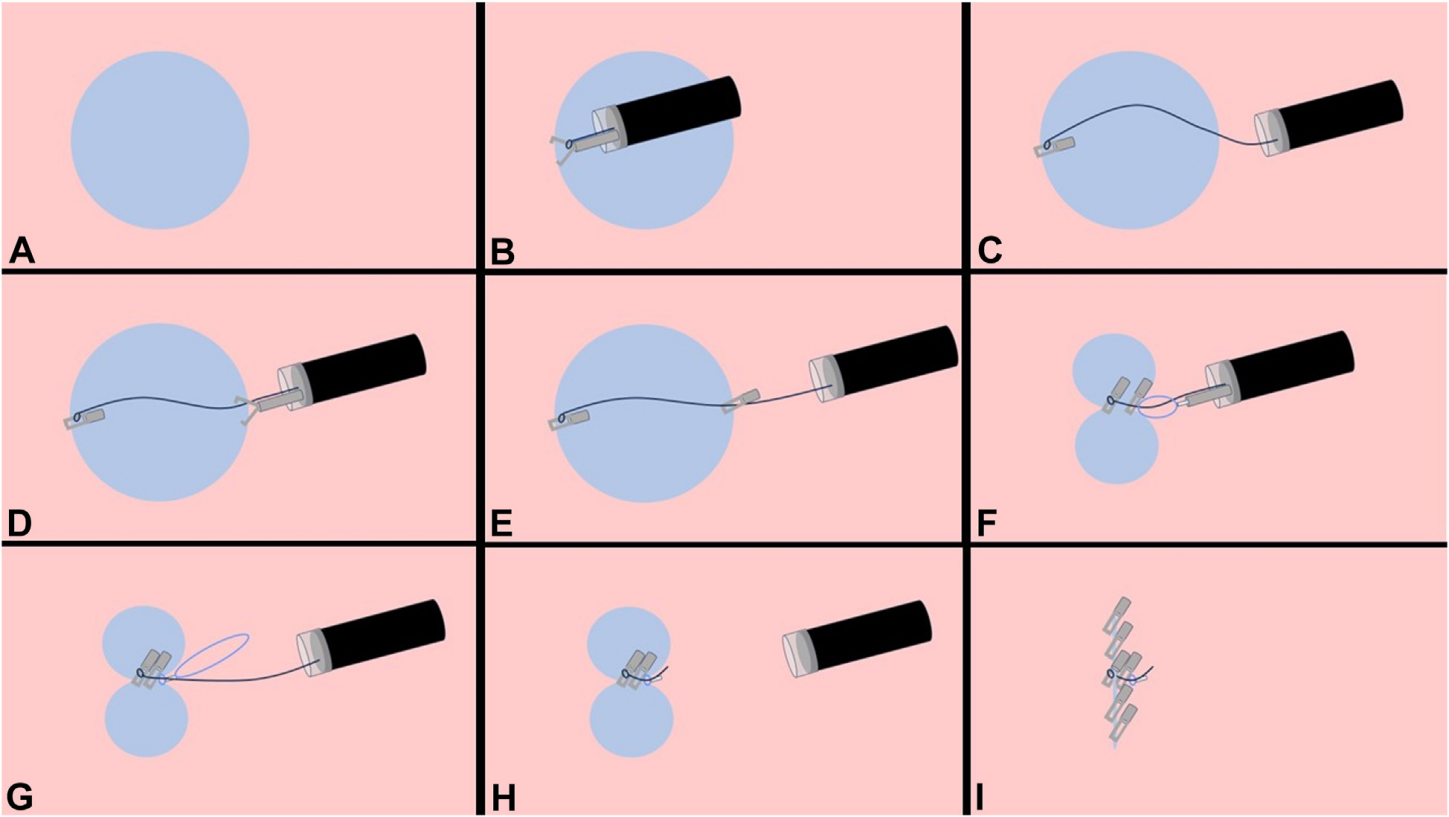
Schematic diagram of clip with line pulley securing technique and a plastic detachable snare. **A,** Mucosal defect after endoscopic submucosal dissection. **B,** A clip with a string is placed at the distal margin of the mucosal defect. **C,** A string is coming out from the instrument channel of endoscope. **D,** A second clip is hooked onto the string. **E,** A second clip is placed at the proximal side of the margin of the mucosal defect. **F,** A plastic detachable snare is inserted along the string through an instrument channel. **G,** Tighten with a plastic detachable snare and a plastic detachable snare is deployed to form a knot. **H,** The string and plastic detachable snare are cut with a loop cutter. **I,** Additional endoscopic clips are placed to achieve complete closure.

In this technique, a clip (Sure Clip; Micro-Tech, Nanjing, China) (Fig. 1A) with a string (Bear nylon monofilament suture; BEAR Medic, Ibaraki, Japan) (Fig. 1B) is placed at the distal margin of the mucosal defect. A second clip is then hooked onto the string and placed at the proximal side of the margin. Both sides of the mucosal defect are gathered by pulling the free end of the string (Video 1, available online at www.videogie.org). Subsequently, a plastic detachable snare (Polyloop; Olympus, Tokyo, Japan) (Fig. 1C) is inserted over the string through the instrument channel. After the edges are approximated well enough, the plastic detachable snare is deployed to form a knot. The free ends of the string and the plastic detachable snare are cut with a loop cutter (FS-5L-1; Olympus) (Fig. 1D). The approximation of the edges of the defect achieved by this technique allowed for subsequent completion of closure with addition of through-the-scope (TTS) hemostatic clips. The technical steps are summarized in Figure 2.

## Case 1

**Figure 3:**
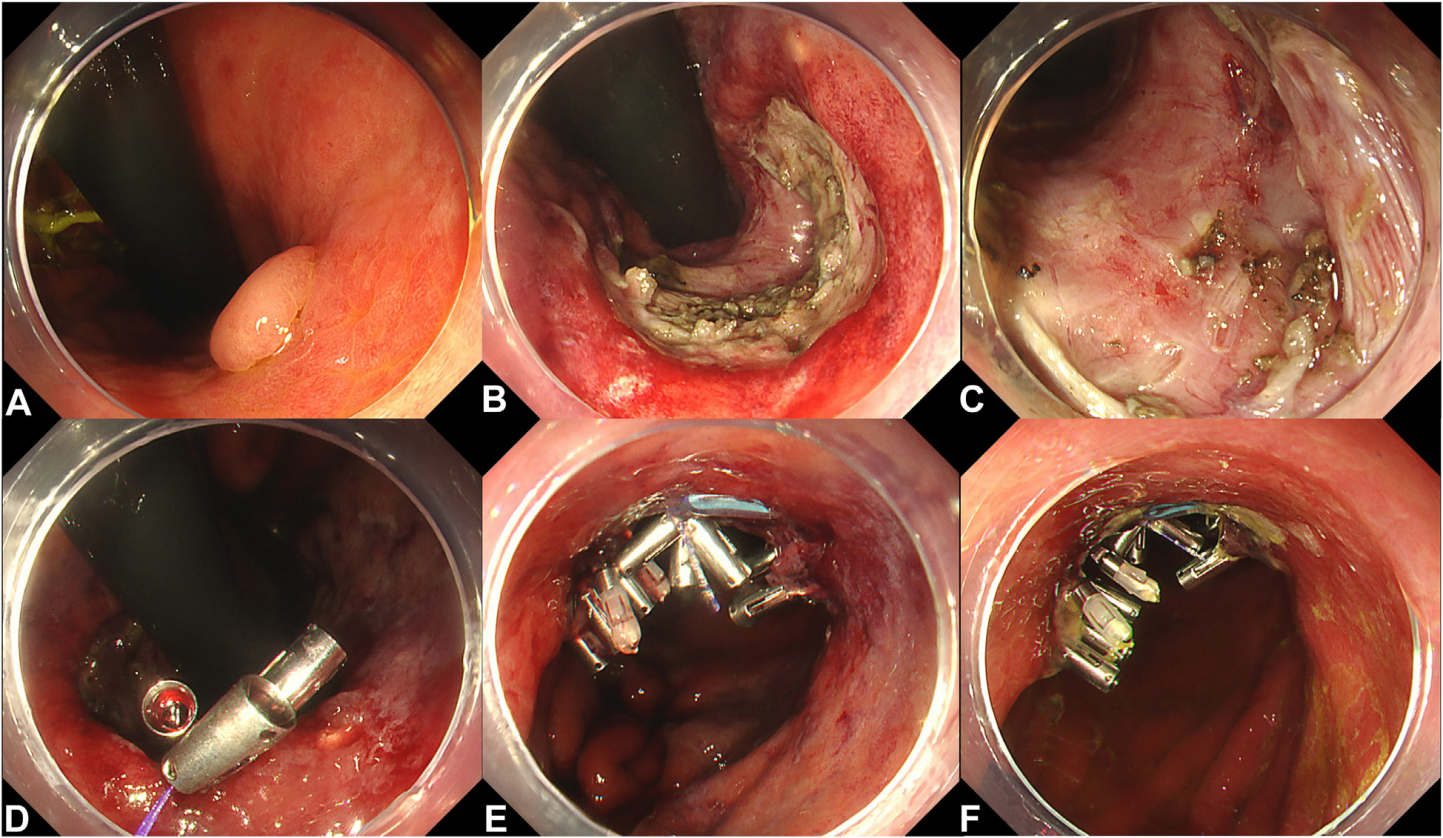
Closure of gastric endoscopic submucosal dissection (ESD) defects using clip with line pulley securing technique and a plastic detachable snare. **A,** Lesion located in the lesser curvature of the middle body. **B,** Ulcer floor after gastric ESD. **C,** Muscular layer injury in ulcer floor. **D,** Both sides of the gastric mucosa of the mucosal defect were gathered, and tension was applied to the string. **E,** Completely closing the ulcer floor. **F,** The sustained closure on postoperative day 3.

An 85-year-old man underwent treatment for a 10-mm early gastric cancer in the lesser curvature of the middle body (Fig. 3). Because of the risk of a thromboembolic event, gastric ESD was performed without the interruption of anticoagulant therapy. The resultant defect was approximately 3 cm in size. Due to slight muscular layer injury, and to prevent subsequent perforation and bleeding, the defect was closed. The CLiPS 3.0 technique was used to approximate the edges of the defect first, followed by application of additional TTS clips to complete the closure. The closure process was completed in approximately 15 minutes. Follow-up endoscopies on postoperative day (POD) 3 confirmed sustained closure. The patient was discharged 5 days after the procedure, with no adverse events.

## Case 2

**Figure 4:**
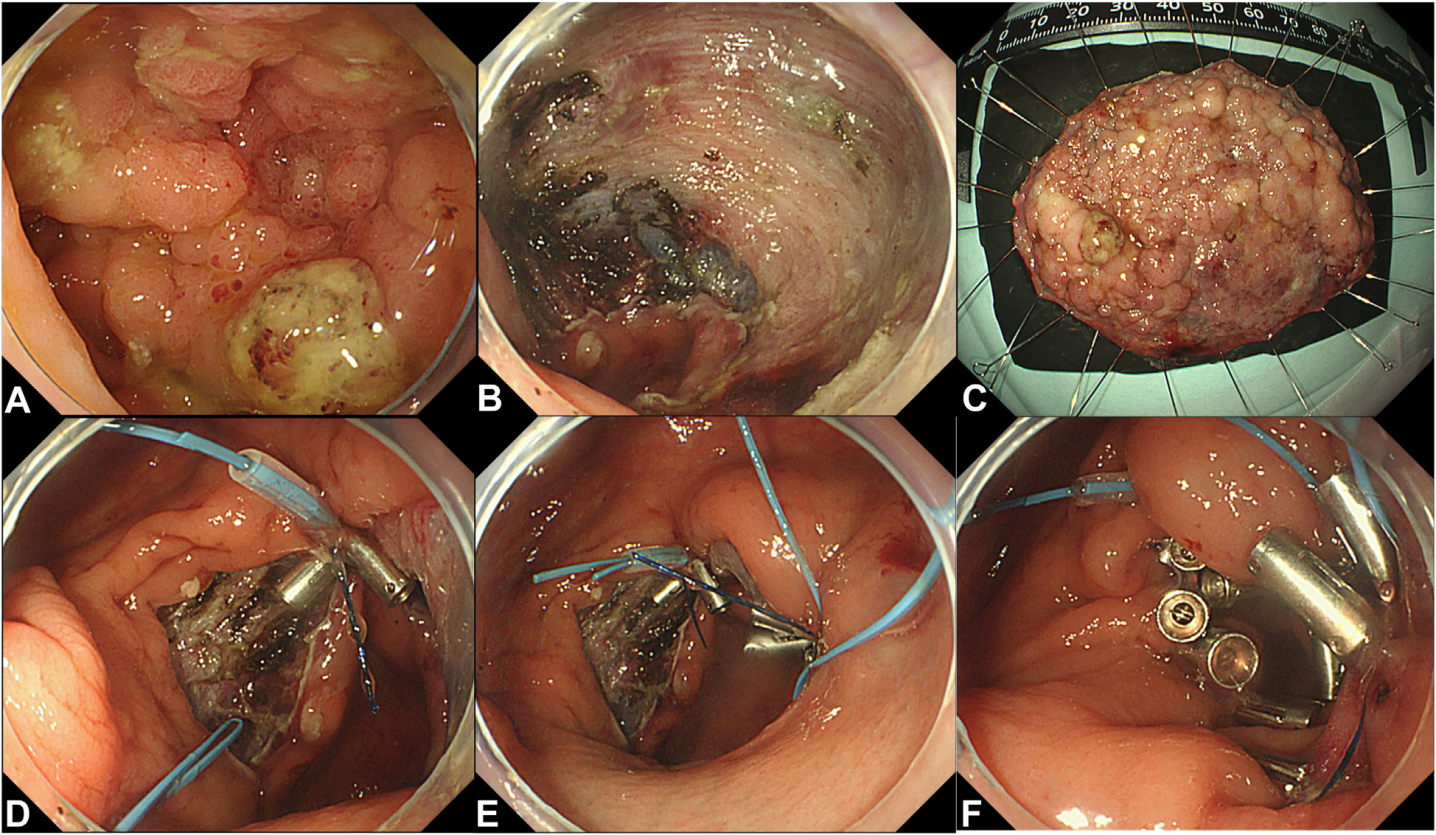
Closure of colorectal endoscopic submucosal dissection (ESD) defects using clip with line pulley securing technique and a plastic detachable snare. **A,** Lesion located in the cecum. **B,** Ulcer floor after colorectal ESD. **C,** Resected specimen; en bloc resection was achieved. **D,** The first clip and string were tightened with a plastic detachable snare. **E,** The second clip and string were tightened with a plastic detachable snare. **F,** Completely closing the ulcer floor.

An 85-year-old woman underwent ESD for an 80-mm early colorectal cancer in the cecum (Fig. 4). The post-ESD defect required 2 iterations of the CLiPS 3.0 technique. Following approximation of the edges of the mucosal defect, complete closure was attained with the use of additional TTS clips. The closure could be completed in 25 minutes. The patient was discharged 3 days after the procedure without any adverse events.

## Discussion

In case 1, we introduce the successful closure of a large defect with the CLiPS 3.0 technique and demonstrate its short-term integrity by endoscopy on day 3 (Table 1). In case 2, we demonstrate the successful application of 2 consecutive iterations of the same technique to close a very large defect.

**Table 1:** Characteristics and outcomes of CLiPS technique

No.	Age	Sex	Lesion size (mm)	Specimen size (mm)	Time for ESD (min)	Complete defect closure	Time for CLiPS technique (min)	Sustained closure	Post-ESD adverse events
1	85	M	10 × 8	27 × 13	30	Yes	15	Yes (POD 3)	None
2	85	F	81 × 59	85 × 63	80	Yes	25	Unconfirmed	None

*CLiPS*, Clip with line pulley suturing; *ESD*, endoscopic submucosal dissection; *F*, female; *M*, male; *POD*, postoperative day.

The fundamental essence of the CLiPS technique lies in its core principle: the reduction of defect size through a robust approximation of the edges. What sets our technique apart is its simplicity in execution: it demands no extra accessories, specialized endoscopes (no need for a double channel scope), or scope exchange. Although the version of CLiPS technique we describe here (CLiPS 3.0) is different from the previous versions we described, each version of the CLiPS technique has its own strengths and limitations (Table 2), making each of these relevant depending on the situation. This technique may even be suitable for closure after full-thickness resections.

**Table 2:** Overview of features in CLiPS versions

Technique	Clip	Line	Pulley	Securing system	Pros	Cons
CLiPS with modified anchoring clip (CLiPS 1.0)	TTS clips	1 line	Second TTS clip	Manually modified TTS clip	Can be used in situations in which plastic detachable snare is not available	Requires manual modification of clips
CLiPS with 2 lines and plastic detachable snare (CLiPS 2.0)	TTS clips	2 lines	Plastic detachable snare	Plastic detachable snare	Useful in instances in which the scope approaches perpendicular to the defect and maneuverability is limited	1. Expensive owing to price of plastic detachable snare 2. Requires 2 lines 3. Not suitable in tangen-tial approach
CLiPS with 1 line and plastic detachable snare (CLiPS 3.0)	TTS clips	1 line	Second TTS clip	Plastic detachable snare	1. Does not require manual modification of clips 2. Does not require 2 lines	1. Expensive owing to price of plastic detachable snare

*CLiPS*, Clip with line pulley suturing; *TTS*, through-the-scope.

We could confirm sustained closure on POD 3 in 1 case (case 1) alone, and the duration of suture maintenance beyond POD 3 remains unconfirmed. This remains a limitation of this study. Larger studies are necessary to assess technical and clinical outcomes of this technique.

## Conclusion

The version of the CLiPS technique demonstrated here is a robust method to close large defects. Multiple iterations of the technique may be used for the closure of very large defects.

## Disclosure

The authors disclosed no financial relationships relevant to this publication.

## Supplementary data

Video 1 Clip with line pulley securing technique and a plastic detachable snare for endoscopic submucosal dissection closure.
